# Policy entrepreneurs in international river basins—getting climate adaptation on the cross-border water policy agenda

**DOI:** 10.1007/s10113-017-1178-5

**Published:** 2017-07-21

**Authors:** Tobias Renner, Sander Meijerink

**Affiliations:** 1 Institute for Science, Innovation and Society, Radboud University, P.O. Box 9010, 6500 GL, Nijmegen, The Netherlands; 2 Institute for Management Research, Radboud University, PO Box 9108, 6500 HK, Nijmegen, The Netherlands

**Keywords:** Rhine delta, Policy entrepreneurs, Regional river basins, Cross-border climate adaptation, Transboundary cooperation

## Abstract

In this study, we look at the role which water policy entrepreneurs play in promoting and stimulating climate adaptation measures in international river basins. In a Dutch-German case study in the Rhine delta, we explore the range of strategies that policy entrepreneurs employ in cross-border water management to effectively anchor and embed climate adaptation in the water policy debate.

We focus on climate adaptation on the local and regional scale in the Deltarhine region where increased flooding and prolonged drought periods are expected under the current climate change scenarios with a considerable impact on flood protection, agricultural activities, drinking water and ecosystem development.

We analyse the impact of policy entrepreneurs while coping with the challenging cross-border setting and dealing with structural differences in national systems such as the legal and institutional framework. It is shown that whilst the European water guidelines advocate a river basin approach across borders, the guidelines do not (yet) play a catalyst role regarding climate adaptation, and the presence and activities of policy entrepreneurs contribute in putting climate adaptation on the cross-border policy agenda.

Finally, marked differences in the presence of entrepreneurs in Germany and the Netherlands are observed for which two important complementary explanations are offered relating to contextual elements of power asymmetry and dependency as well as different policy styles and organisational cultures in both countries.

**Electronic supplementary material:**

The online version of this article (doi:10.1007/s10113-017-1178-5) contains supplementary material, which is available to authorized users.

## Introduction

### Setting the scene

The water sector is arguably one of the most important sectors in which climate adaptation efforts are concentrated as the hydrological cycle will be severely impacted by a changing climate. The future water situation and developments in the water sector have been examined by the Intergovernmental Panel on Climate Change (IPCC) under different climate change scenarios and point to increased vulnerability to water scarcity, droughts and floods (IPCC 2014).

Scenario studies on the effects of climate change commissioned by the International Commission for the Protection of the Rhine (ICPR 2011) show a considerable decrease of average precipitation in the Rhine catchment for the second half of this century during the summer period (reduction 10–30%) with a comparable decrease of low flow discharges. At the same time, an increase of extreme rainfall events is expected, leading to heightened risk of serious flooding along the Rhine. This paper describes a Dutch-German case study in the delta of the Rhine catchment focusing on the role of policy entrepreneurs in promoting climate adaptation. We focus on the local and regional scale where increased flooding and prolonged drought periods are expected under the current climate change scenarios with a considerable impact on flood protection, agricultural activities, drinking water supply as well as ecosystem development.

**Fig. 1:**
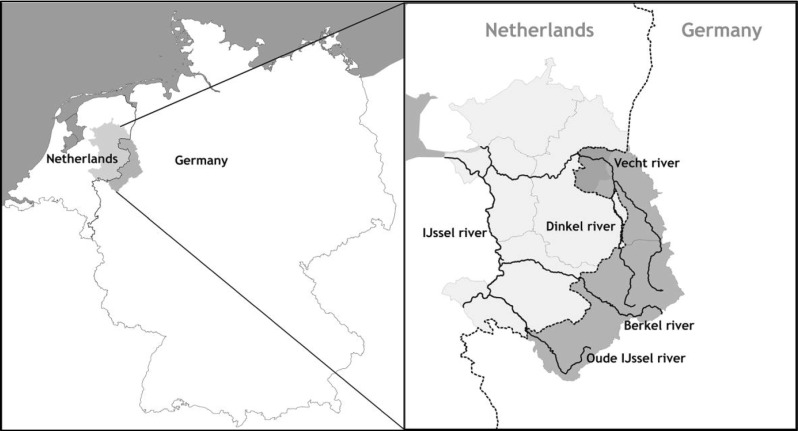
Overview map of the study area

Water does not stop at administrative or jurisdictional borders, and in international river basins such as the Rhine, a river basin approach as advocated by the European Water Framework Directive is needed, where riparian countries work together to adapt to a changing climate. The Deltarhine region is one of the nine international sub basins in which the Rhine basin has been subdivided under the European Water Framework Directive (IRBM Rhine 2009). It is the most downstream sub-basin of the Rhine and is shared by Germany and the Netherlands, with the Netherlands being the downstream country. In this study, we look in particular at the smaller cross-border shared river systems in Deltarhine, such as the Vecht, Dinkel, Berkel and Oude IJssel river (Fig. 1).

Cross-border policy making in the Rhine basin essentially takes place in what Durth (1996) has called an integrated environment, with the two neighbouring countries, Germany and the Netherlands, having similar cultural roots, a common historical background and a joint supranational European legal and institutional framework.

Nevertheless, various authors have convincingly shown (Keetman 2006; Van Leussen et al. 2007; Wiering et al. 2010; Van der Molen 2011; Van Eerd et al. 2015; Jacobs 2016), which cross-border water policy making in the Dutch-German context still has to deal with marked differences in the respective institutional and legal framework, with different ambition levels and discourses regarding climate change and climate adaptation, with asymmetries due to the upstream-downstream relationship in the international river basins as well as cultural differences and language barriers between both countries.

Van Leussen et al. (2007) have given a detailed description of the institutional and political context in the study area, providing a detailed account of the institutional differences and similarities between Germany and the Netherlands. In short, there are marked different institutional structures in the two countries, leading to institutional mismatches between hierarchical levels in both countries and providing a challenge for regional cross-border cooperation. Within Germany, the institutional setting is further complicated by the fact that two federal states, Lower Saxony and North-Rhine Westphalia with a different institutional structure, are involved in the study area. In total, there are nine Dutch and German water authorities at the national and regional level directly involved in regional water policy making and climate adaptation efforts in the study area.^1^ In general, the German institutional water management structure in the study area is more fragmented, especially with regard to implementation, and has no equivalent to the Dutch regional water authorities (waterboards) which have a unique degree of freedom and mandate in policy making and implementation at the regional level (Keetman 2006).

On the regional scale of Deltarhine, increased frequency and severity of floods and droughts stemming from climate change are seen as the greatest potential threats to a robust and sustainable integrated management of the shared water resources. Accordingly, suggested policies seek to address the issues of increased likelihood of floods and droughts (water shortage) through a variety of measures, both traditional such as strengthening of dikes, as well as relatively new concepts such as giving more space to the river in combination with river restoration and novel approaches such as ecosystem-based adaptation strategies.^2^ Sustainable groundwater management and future potential threats associated with climate change to groundwater abstraction take a distant third place as issue area in connection with climate adaptation. In the study area, we find that seven particular venues of cross-border cooperation were used to introduce climate adaptation into the cross-border policy debate and to jointly address the abovementioned issues. For an overview of the venues, see Table 1 below, while a non-exhaustive overview of corresponding regional policy documents is provided in Table 3.^3^

**Table 1:** Venues of cross-border cooperation dealing with climate adaptation

Venues of cross-border cooperation dealing with climate adaptation	Time period	Description
Climate adaptation project KARMA	2013–2015	Cross-border initiative dedicated to climate adaptation and promoting Ecosystem based Adaptation Strategies
Cross-border Platform for Regional Water Management	2012–2016	Regional cross-border platform addressing climate adaptation challenges (flooding, water shortage)
Cross-border project Climate adaptation in the Dutch-German Dinkel river	2010–2014	Cross-border project dedicated to climate adaptation and river restoration (*Veerkrachtige Dinkel*)
Cross-border Vechtvision	2007–2009	Cross-border spatial development scheme, addressing climate adaptation through restoring the natural retention capacities
Cross-border restoration project Schoonebeekerdiep	2006–2015	Cross-border project to address river restoration and climate adaptation
Cross-border project ‘Gewässerkonzept Schlinge’	2013–2015	Cross-border project to address flood protection, land consolidation and climate adaptation
SGDR/AGDR – Cross-border Coordination Water Framework Directive	2006–2015	Cross-border coordination of WFD river basins management plans, addressing climate change

### Focus of this study

In this study, we focus on the question what, if any role policy entrepreneurs play in this challenging cross-border setting to overcome the abovementioned barriers to cooperation, joint policy making and action on climate adaptation. We specifically concentrate on the local and regional aspects of cross-border cooperation regarding climate adaptation. Regional actors in border areas are crucial to develop and implement climate adaptation policies and are directly confronted with the challenges of cooperation as well as any inconsistencies and differences in national policies regarding climate adaptation. Regional and local authorities play a decisive role in establishing cross-border cooperation in smaller shared river systems (Jacobs 2016), and with regard to climate adaptation, we analyse policy entrepreneurship at this level. With very few exceptions, scholars of cross-border cooperation the water domain have not focused on the role of key individuals and vice versa, the concept of policy entrepreneurship has, as yet, been hardly applied in a regional cross-border setting. With this study, we thus seek to contribute to both the literature on entrepreneurship in climate adaptation and on cross-border cooperation in international, shared river basins.

Focusing on the presence, activities and finally the impact of policy entrepreneurs, we formulate our central research question as follows: *What is the impact of entrepreneurial activities on climate adaption in the shared river basins in Deltarhine?*

## Analytical framework and methodology

### Analytical framework

Policy entrepreneurship in the study area is analysed by applying and operationalising an analytical framework with four main components to answer the following detailed research questions on emergence, strategies and impact of entrepreneurs as well as the cross-border context within which they operate: (1) presence—which key individuals, entrepreneurs can we identify, who are trying to shape cross-border climate adaptation in the study area? (2) activities—what range of entrepreneurial strategies do they use to overcome cross-border differences and promote climate adaptation? (3) impact—are the entrepreneurial efforts and activities indeed leading to policy change, both on paper and on the ground? And finally (4), what differences, if any, do we see in entrepreneurship between the Netherlands and Germany and what explanations can be found in contextual variables in both countries (i.e. different cultural or institutional setting)?

#### Identifying and characterising individual policy entrepreneurs

Policy entrepreneurs are generally described as highly motivated individuals who advocate issue-specific policy changes and try to get policy solutions and measures on the governmental agenda (Roberts and King 1991; Kingdon 1995; Mintrom and Vergari 1996; Mintrom and Norman 2009). Cross-border policy entrepreneurs are individuals who want to initiate or stimulate cross-border cooperation in specific issue areas, in our case climate adaptation in international river basins, and seek to gain attention for issues, try to get these issues on the cross-border policy agenda and to seek adoption of and support for policy change and policy measures, both on paper and on the ground. General characteristics of entrepreneurs are their willingness to invest their resources (time, energy, reputation and/or knowledge) in particular policy efforts, possessing good networking skills and demonstrating considerable perseverance (Kingdon 1995; Meijerink and Huitema 2010; Mintrom and Norman 2009; Roberts and King 1991), and these characteristics are put to use in this study.

#### Investigating strategies employed by policy entrepreneurs

Policy entrepreneurs use variegating sets of strategies to spread ideas (Mintrom and Norman 2009; Meijerink and Huitema 2010; Jordan and Huitema 2014a, b). We are interested to analyse how policy entrepreneurs cope with challenges and barriers to push climate adaptation on the cross-border policy agenda, while dealing with structural differences in national systems such as the legal and institutional framework. In this study, we use the approach which was developed by Huitema and Meijerink (2009) and explore four main strategies (1) framing and idea development to deal with different cross-border discourses and legal requirements, (2) recognition, exploitation and manipulation of venues to deal with differing institutional and organisational framework in Germany and the Netherlands, (3) the recognition and exploitation of windows of opportunity, such as flood events, and (4) the orchestration and management of cross-border networks to deal with cultural differences and language barriers.

#### Analysing effects of entrepreneurial activities

Intractable, complex policy challenges such as climate adaptation are not easily amenable to policy changes, and even if there are policy changes on paper, being adopted and prescribed in policy documents, this not easily translated into changes on the ground (Huntjens 2011; Termeer et al. 2013). This holds also true for the water sector where climate adaptation measures such as strengthening dikes or creating large-scale water retention areas requires the availability of land with an impact on other stakeholders in the policy domains of spatial planning, agriculture, energy supply or infrastructure. Naturally, this touches also on the wide field of implementation research and in particular the challenge to implement policies to jointly solve such problems in the international arena with its main characteristic of absence of central authority. In this study, we will make use and apply a conceptual framework developed by Jordan and Huitema (2014a). As they noted in their paper on policy innovations on climate adaptation, policy change as a general and undifferentiated concept can be a rather blunt category robbing it of explanatory value. Drawing on well-established perspectives on policy innovations, they develop a new analytical framework based on a more holistic approach by distinguishing between three concepts, which we will put to use in this study: (a) the invention of policies (and elements therein) that appear to be new, through the activities of ‘policy entrepreneurs’, (b) policy diffusion (transfer, lesson drawing) and adaptation to local circumstances and (c) policy impact (emerging impacts, i.e. do they have significant and long-lasting impacts).

#### Contextual variables and differences in entrepreneurship between Netherlands and Germany

Though is not the main focus of this study, we will also explore what any differences we can find between entrepreneurship in both countries. Mintrom (1997) showed how policy entrepreneurship could be studied systematically and demonstrated that the likelihood of policy change is affected by key contextual variables and by what policy entrepreneurs do within those contexts (Mintrom and Vergari 1996; Mintrom and Norman 2009). Mintrom has suggested executing cross-national studies of policy entrepreneurship to leverage the study of policy entrepreneurship in new contexts so as to achieve conceptual breakthroughs. While realising the limited geographical scope of our study, we will be on the lookout for contextual factors that might explain observed differences in Dutch and German entrepreneurship.

### Methodology

This case study analysis is firstly based on an examination of archival records, minutes of meetings, secondary literature and relevant policy documents from Dutch and German water authorities as well as cross-border organisations and institutions in the period between 2005 and 2015. In particular, the policy documents listed in Table 3 have been examined and analysed, as they are directly linked to the identified venues of cross-border cooperation dealing with climate adaptation in the study area. In the Electronic Supplementary Information, an extended overview (Table S1) is provided of the archival records that were consulted; in Table S2, an overview of literature and publications about the study area is provided.

Second, the analysis was complemented with information from semi-structured interviews with in total 20 respondents (10 from Germany, 10 from the Netherlands), mostly from the regional and local level. The interviews were carried out in 2013 and 2014 with main questions pertaining to (a) the current and future policy challenges in cross-border cooperation concerning climate adaptation related issues such as flood protection and water shortage, (b) main drivers of cross-border cooperation in these issue areas, (c) role and activities of key individuals, and their impact (d) policy changes on paper and on the ground and (e) contextual variables (i.e. institutional, organisational, cultural setting). The interviews were open, semi-structured and held in German and Dutch to make it easier for the respondents to express nuances and detailed descriptions in their own language. Table S1 and S3 Electronic Supplementary Information provide additional information on the interviews.

Third, participant observation was used, with the lead author supporting Dutch-German projects in the study area since 2007, and in particular the Dutch-German Cross-border Platform for Regional Water Management between 2012 and 2015 as well as the KARMA project from 2013 onwards.

The abovementioned sources of information were jointly used to triangulate information and findings, in particular regarding the presence, activities and impact of policy entrepreneurs.

## Findings

### Entrepreneurship in the study area

Key individuals can indeed be identified in the study area who employ different strategies to insert climate adaptation into the cross-border policy debate. These key individuals can be described as water policy entrepreneurs who play a pivotal role in pushing the climate adaptation related issues on the regional water policy agenda. Limited in number, they share the general characteristics of policy entrepreneurs such as a persevering ability and willingness over long periods of time, in the order of at least several years, to put climate adaptation repeatedly on the policy agenda of different cross-border institutions and venues for Dutch-German cooperation and information exchange. The policy entrepreneurs were identified by analysis of policy documents and archival records, from participant observation as well as from interviews, where a consensus emerged on individuals that were seen as key to pushing the issue of climate change adaptation and related measures forward, while not being formally obliged or expected to do so. They are displaying the characteristics of policy entrepreneurs as described in section “Analytical framework”, which sets them apart from the larger group of about 60 Dutch and German policy makers and experts, involved in cross-border cooperation in the study area. The identified policy entrepreneurs (which were also interviewed) are civil servants at senior level mostly from regional Dutch authorities, dealing with water management, ecosystem preservation and nature conservation, such as waterboards and provinces. The skewed distribution of entrepreneurial activities towards entrepreneurs from the Netherlands (see Table 2 below) is duly noted and further explored below in section “Differences in Dutch and German entrepreneurship”.

**Table 2:** Identified policy entrepreneurs in the study area

PE positioned at organisation	Position	Nationality	Climate adaptation measures regarding	Involved in cross-border venues
Waterboard Velt en Vecht	Civil servant	Dutch	Flood protection, room for the river in combination with river restoration	Cross-border Platform for Regional Water Management • Cross-border restoration project Schoonebeekerdiep • Cross-border Vechtvision
Waterboard Regge en Dinkel	Civil servant	Dutch	Flood protection in combination with river restoration	• Cross-border Platform for Regional Water Management • Cross-border project Climate adaptation in the Dutch-German Dinkel river
Province of Overijssel	Civil servant	Dutch	Water shortage, sustainable groundwater management	• SGDR/AGDR – Cross-border Coordination Water Framework Directive
Province of Gelderland	Civil servant	Dutch	Flood protection, water shortage, and retention, ecosystem-based adaptation	• Climate adaptation project KARMA • Cross-border Platform for Regional Water Management • SGDR/AGDR – Cross-border Coordination Water Framework Directive
Bezirksregierung Münster	Civil servant	German	Flood protection, ecosystem-based adaptation	• SGDR/AGDR – Cross-border Coordination Water Framework Directive • Cross-border project ‘Gewässerkonzept Schlinge’

Furthermore, we found no evidence that entrepreneurs from non-governmental, private organisations or the scientific community have as yet become actively involved in climate adaptation efforts across borders. These findings are partly in line with the earlier studies of Becker and Huitema where it was found that civil servants and experts within the government as well as the scientific community have played a major role, which they explain by the highly technical nature of the water sector (Becker 2009; Meijerink and Huitema 2010). We would like to add the observation that international river basin management and cross-border cooperation seem to be firmly rooted in the public domain due to its public good character and with sovereignty and foreign policy issues playing up in border regions, even at the regional and local level.

### Activities—entrepreneurial strategies employed in cross-border cooperation

How, then, do policy entrepreneurs actually try to effect policy change, how do they go about putting climate adaptation on the international agenda and work towards policy innovation, diffusion and finally implementation of measures such as pilot projects. What strategies did they use to overcome institutional barriers?

To start with, it is necessary to state that we encountered only one example of a reflective practitioner, a cross-border policy entrepreneur choosing and using consciously different strategies. That is not to say that other key individuals did not employ specific strategies; however, they were more skilled and in favour of a ‘trial-and-error’ approach instead of a conscious reflection on available strategies to for example create, orchestrate and structure cross-border inter-organisational issue networks around climate adaption. That said, the identified policy entrepreneurs employ consciously or unconsciously a range of strategies to advocate climate adaptation in the water sector while dealing with structural institutional, legal and cultural differences between Germany and the Netherlands as well as existing language barriers, which are described below using the approach suggested by Huitema and Meijerink (2009).

#### Framing and idea development to deal with different cross-border discourses and legal requirements

Climate adaptation has been introduced into the policy debate in the study area by processes of both policy invention and diffusion. The notion of Jordan and Huitema (2014a, b) that policy invention can be a restrictive category, if interpreted as ‘newness’ on a global scale, and can lead to focus more broadly on inventive activities of policy entrepreneurs, certainly applies on the regional scale of Deltarhine. There have been examples of what we might regard as policy invention in the early stages of agenda setting and policy formulation, such as attempts at setting up a climate adaptation masterplan for the Dinkel and introduction of the ecosystem based adaptation strategies, attempts which are certainly inventive and innovative in the sense that they are new to the study area. However, these efforts by policy entrepreneurs also contain strong elements of policy diffusion and policy learning from other European and Dutch-German initiatives. The 2000s have seen the introduction of ideas and policy proposals on climate adaptation as evidenced for example by corresponding chapters and references in policy documents such as the WFD International River Basin Management plan 2009–2015.

The domestic policy debates between Germany and the Netherlands differ on the regional governmental level of districts, Dutch waterboards, provinces and the German regional authorities (*Bezirksregierungen*). Climate adaptation in the Netherlands has over the past 10 years been regularly framed as being firmly in the water domain, while in the German policy debate efforts at the regional level, efforts have centred on mitigation in the energy domain and until now only to a limited extent on adaptation the water sector (and then mainly on urban planning). It has been one of the challenges of the policy entrepreneurs, mostly of Dutch origin, to gain attention for climate adaptation measures regarding flood protection and water shortage on the German side, for example by venue shopping and manipulation.

#### Recognition, exploitation and manipulation of venues to deal with differing institutional and organisational framework in Germany and the Netherlands

The institutional and organisational landscape in the water sector is quite fragmented across the borders with different governmental hierarchies in Germany and the Netherlands and subsequently different roles, responsibilities and mandates of the involved governmental institutions. Within this landscape, a substantial number of cross-border cooperation processes, both formal and informal, have been established in the past few decades, which can best be characterised as governance or inter-organisational networks bridging the border and linking and connecting the differing German and Dutch institutional structures.

The policy entrepreneurs are found to actively use these different venues of cross-border cooperation and also switching between governmental levels from the national level (the so-called SGDR/AGDR structure) over the regional level (Cross-border Platform Regional Water Management-GPRW) down to the local level at the scale of individual watercourses to put climate adaptation on the cross-border policy agenda. Venue manipulation and venue shopping have in particular being observed in recent efforts starting in 2013 to initialise a cross-border project, called KARMA, with European co-funding and using the different cross-border institutions to generate attention, publicity and commitment. Another example concerns long-running efforts to develop a cross-border masterplan for climate adaptation in the Dinkel river, where different venues, through bilateral contacts and the cross-border WFD cooperation structure (SGDR/AGDR), are used.

#### Recognition and exploitation of windows of opportunity such as flood events

It is interesting to note that all identified policy entrepreneurs are aware of the catalytic role that focusing events (Brouwer and Huitema 2016, in this special issue) and windows of opportunities (Kingdon 1995) can play in launching ideas, articulating policy proposals, generating political momentum and mobilising resources. Successful exploitation of problem windows after flood events has occurred in the study area, most notably after flood events in 1998 and 2010 in the Vecht and Dinkel rivers, however not with reference to flood protection framed as climate adaptation measures. Public and political attention has not yet been raised to a level, where climate adaptation can progress from policy formulation on paper, as evidenced by existing (cross border) policy documents, to policy measures on the ground, but policy entrepreneurs are certainly aware that problem or political windows are useful in that regard and can be exploited. As one respondent formulated “Only a severe drought may raise awareness in the short-term that we must start now to address expected future water shortages due to climate change in the Rhine basin in the long term also in the local and regional sub-catchment of Deltarhine”. Another kind of window of opportunity is represented by regular calls for proposals from the European funding programme INTERREG, dedicated to stimulate cross-border cooperation in border regions across Europe. Policy entrepreneurs are seeking to use this funding programme to complement financial resources and to carry out cross-border climate adaptation projects. Policy entrepreneurs were also found to have been actively influencing the Operational Programme of current INTERREG Va programme, by successfully submitting a position paper to include and explicitly mention climate adaptation in the funding guidelines for cross-border cooperation.

#### The orchestration and management of cross-border networks

Cross-border cooperation in the regional shared river systems of Deltarhine is mainly structured and organised in a number of inter-organisational cross-border networks. Orchestrating and steering these cross-border networks is a deliberate attempt at network management undertaken by policy entrepreneurs, that is, to govern these processes and guide the interaction between the Dutch and German actors. Various management strategies have been identified and described in the literature on inter-organisational network management (Kickert et al. 1997; Meier and O’Toole 2001; Huxham and Vangen 2005; Provan et al. 2007), which in short can be categorised either as strategies of institutional design (network structuring) or process management (playing the game). Given this broad categorisation, lumping detailed entrepreneurial strategies,^4^ it is not altogether surprising that these two types of network management strategies have indeed been employed by most of the policy entrepreneurs. However, we observe that there are especially two venues where a broad range of network management strategies have been employed, the KARMA project and attempts to initiate a cross-border planning process for the Dinkel river. In both cases, policy entrepreneurs have taken up themselves the explicit role of process or network manager, paying attention to orchestrating and managing the cooperation process.

In initiating, structuring and managing cross-border networks, policy entrepreneurs have to deal with specific characteristics of the cross-boundary setting, with structural differences on both sides of the border (Jacobs 2016). They use what may be referred to as ‘bridging strategies’ to cope with institutional, organisational, legal and cultural differences between both countries. We observe that network structuring and selective (de)activation of actors in cross-border networks are an important strategy to take different institutional and organisational mandates as well as legal responsibilities into account. Attention to detail, such as selecting network participants and organisations when creating new venues for cooperation as well as balancing the size of delegations and the number of German and Dutch actors is actively employed and observed, exemplary in this regard are the network structuring efforts for the KARMA project.

### Impact of policy entrepreneurs

Over the past 10 years, the issue of climate adaptation has become, through a process of policy invention and diffusion, increasingly embedded in the cross-border policy debate in Deltarhine. Three indicators for this are the following. First, climate adaptation has increasingly gained attention in the cross-border policy debate in Deltarhine as witnessed by various policy documents, starting from 2006 onwards, that are implicitly or explicitly addressing the issue of climate change and the necessity to further explore, initiate and implement adaptation measures in the water sector. Table 3 provides an overview of policy documents such as the Cross-border Vechtvision, the mandate of the Cross-border Platform for Regional Water Management or INTERREG proposal documents (KARMA). Secondly, climate adaptation is discussed in venues of cross-border cooperation such as working groups and expert groups under the AGDR/SGDR and the Cross-border Platform for Regional Water Management, as shown by minutes of meetings and archival records from the past 10 years. Thirdly, spatial planning and engineering studies must be mentioned such as the Schoonebeekerdiep planning, the project ‘Gewässerkonzept Schlinge’ or the continued efforts for a Dinkelplanning seeking to identify and detail climate adaptation measures addressing flood protection and water shortage in the regional river basins. The key question is to what extent these developments may be solely explained by policy entrepreneurship. This is a complex question to answer as other factors, such as a more general change in the water management discourse, or a convergence of national policies, may be at play here. Therefore, we asked our respondents, active participants in cross-border cooperation processes, how they evaluate the role of key individuals in realising the changes described above. The interviews reveal a broad consensus amongst participants that successes in introducing climate adaptation into the cross-border policy debate should be attributed at least partly to the activities of policy entrepreneurs as described in the previous section—an observation corroborated by examining and tracing contributions of key individuals in archival records (minutes of meetings, internal working paper and workshop proceedings).

**Table 3:** Policy documents with cross-border relevance mentioning climate change/adaptation in Deltarhine 2005–2015

Dutch policy documents based on Delta act Room of the River Vecht 2009–2014 River Regge 2007–2009	Dutch investment programs for climate adaptation measures focusing on flood protection.
Freshwater programme East Netherlands (Zoetwatervoorziening Oost Nederland)	Dutch investment program for climate adaptation focusing on water availability / water allocation.
Dutch regional policy documents Watervision 2030 Waterboard Rijn en IJssel (Watervisie 2030)	Separate texts exploring climate adaptation and the need for cross-border cooperation
Draft WFD management plan Rhine-East 2016–2021 (Ontwerp waterbeheerplan Rijn-Oost)	Separate chapter on climate change and adaptation
German policy documents Lower Saxony Draft WFD management plan 2016–2021	Separate chapter on climate change and adaptation.
German policy documents Northrhine-Westphalia Draft WFD management plan 2016–2021	Separate chapter on climate change and adaptation.
Cross-border policy documents Cross-border Vechtvision (Grensoverschrijdende Vechtvisie 2007–2009)	Climate change explicitly mentioned in ensuring flood protection and restoring the natural retention capacity
International RBMP Deltarhine 2009–2015	Chapter 7 on climate change and adaptation.
Draft International RBMP Eems 2016–2021	Separate chapter in climate change and assessing WFD measures in terms of climate adaptation
Archival records AGDR/SGDR – Factsheets Cross-border Climate change and adaptation (2010–2014)	Jointly discussed, however climate adaptation was not prioritised and working group was not formed.
Operational Programme INTERREG V and Dutch-German position paper on importance climate adapation in the future INTERREG V programme (2012)	Cross-border climate adaptation explicitly in subsidy guidelines of the Dutch-German INTERREG Va Programme (Operationeel progr.)
Study reports and documents	
Cross-border restoration project Schoonebeekerdiep (2004–2014)	Climate change scenario’s used in the Netherlands but not in Germany
Draft Cross-border Interreg-project KARMA 2014	Cross-border project dedicated to climate adaptation and promoting Ecosystem based Adaptation Strategy
Draft Cross-border project Climate adaptation in the Dutch-German Dinkel river (2014)	Cross-border project dedicated to climate adaptation (Veerkrachtige Dinkel)
Cross-border project ‘Gewässerkonzept Schlinge‘(2015)	Cross-border project dedicated to water retention, water shortage and climate adaptation
Major climate change research projects Knowlegde for climate (KiK) – Netherlands KLIMZUG, KLIFWA and Dynaklim – Germany	Major research projects dealing with governance aspect as well as technical aspects (modelling studies, scenario analysis adaptation measures etc.)

However, climate adaptation has become neither a vital nor a central element of the water policy narrative in regional cross-border cooperation. Climate change and adaptation are mentioned in cross-border policy documents as described above, but are not yet translated in coordinated cross-border projects and climate adaptation measures on the ground, as also demonstrated by the KARMA initiative, a proposed INTERREG project dealing with climate adaptation, which stalled in 2015. The necessary next step towards regional implementation and policy evaluation on climate adaptation measures is yet one that has to be taken and is also at the centre stage of the current activities of policy entrepreneurs, i.e. to translate general policy recommendations on climate adaptation into master planning and small-scale pilot projects to demonstrate the feasibility and benefits of the approaches that are advocated. As one respondent remarked “We have now talked for several years about climate adaptation, now we need to do projects”.

Whilst the European water guidelines advocate a river basin approach across borders and the respective policy documents contain clear reference to climate change, they not (yet) play a catalyst role regarding climate adaptation measures in the study area. Rather, entrepreneurial activities have helped pushing and embedding climate adaptation in the cross-border policy agenda; however, the stage of policy implementation and evaluation has certainly not yet been reached. This finding is in line with lessons from other studies, where it is found, that while new policies may be formally adopted, they neither replace existing policies entirely nor are they fully implemented. Formal policies as evidenced by policy documents (policy output) have been changed but implementation constitutes a next step, ‘a new round in the policy game’, where established routines are often less amenable to change (Andresen and Agrawala 2002; Saetren 2005; Meijerink and Huitema 2010). Two complementing explanations are offered for the observed lack of progress and the limited impact and stymied efforts of policy entrepreneurs in moving towards joint and coordinated cross-border actions on climate adaptation on the ground.

First, we observe a different sense of urgency and different policy styles in Germany and the Netherlands regarding climate adaptation. There are national climate adaptation programmes in Germany and the Netherlands, with the Dutch side being clearly ahead in policy formulation and implementation, for example in realising flood protection programmes for the large rivers taking into account IPCC climate change scenarios (space for the river programme). German actors are still more involved in agenda setting and policy preparation, in particular in research efforts to reduce uncertainty in assessing the effects of climate change on the local and regional level (KLIMZUG 2012). This difference in ambition and phasing on how to deal with climate change is also mirrored at the regional level of Deltarhine, with the Dutch side being more active in pushing climate adaptation on the policy agenda.

Second, an additional reason for the observed lack of progress lies within the fact that incremental, technically oriented climate adaptation measures, such as dike strengthening, which are closely within the decision-making sphere of the concerned water authorities, are relatively uncontested. Radical innovations, however, advocated by policy entrepreneurs in the study area, such as the Ecosystem based Adaptation Strategy with broader economic implications, especially for agricultural land use, meet resistance. It should be noted that the German parts of the study area, the Districts of Borken and Landkreis Grafschaft Bentheim, are amongst the most intensively farmed agricultural areas in Germany.

In addition, it is necessary to note that German pioneering and entrepreneurial activities regarding climate mitigation in the energy domain are hampering efforts on climate adaptation in the water domain. The implementation of the Renewable Energy Act in Germany, the “*Energiewende*” in recent years has led to increased production of bio-fuels and intensifying land use in study area which in turn has decreased the willingness of farmers to participate in climate adaptation schemes requiring agricultural lands to near zero. Agricultural lands are increasingly used for intensive farming and to grow energy crops such as biomaïze, reducing the availability of land to realise water retention areas or implement eco-system based approaches in land cultivation (KARMA 2014). Water policy entrepreneurs are thus encountering hard economic boundaries, created by policy incentives, related to climate change mitigation in the energy domain, to further promote and implement climate adaptation measures in the water policy domain.

### Differences in Dutch and German entrepreneurship

While not carrying out a comparative Dutch-German analysis, we also looked for obvious differences between Dutch and German entrepreneurship. When looking at the presence, activities and impact of entrepreneurs we found as most prominent and significant difference, a skewed distribution of entrepreneurship, with most entrepreneurs at the regional and local level coming from the Netherlands, for which three complementary explanations are offered. First, the upstream-downstream relationships introduces an element of asymmetry and dependence for the downstream, regional Dutch water authorities, resulting in a more active role in cross-border cooperation on the Dutch side. Second, there are indications that different policy styles and organisational cultures may play a role, with a governance-oriented public water sector in the Netherlands being more enabling for policy entrepreneurs than the more hierarchically organised German water sector, especially at the local and regional level. This explanation is in line with the study by Keetman (2006) applying the model from Hofstede (1991) in analysing cross-border cooperation in Deltarhine and Steers et al. (2013) who amalgamates different cultural (organisational) theories and compares the Anglo-saxon and Germanic organisational cultures with criteria such as power distribution, social and environmental relationships, work patterns and social control. The enabling character of the Dutch water sector for policy entrepreneurs, which seems to contrast with a German public administration more in line with a Weberian ideal, has also been described in the work of Brouwer (2013) on Dutch water policy entrepreneurs. Third, it is observed that the institutional landscape in the German federal states with regard to water management is fragmented, and in the study area, there is no institutional complement to the Dutch water authorities, who are dedicated to regional water management and might provide an enabling environment for policy entrepreneurs with substantial personnel and financial resources (Van Leussen et al. 2007).

## Discussion and conclusions

Returning to our main research question on the impact of entrepreneurial activities on cross-border climate adaption in the shared river basins in Deltarhine and summarising our results, we find that key individuals can indeed be identified who employ a range of strategies to insert climate adaptation into the cross-border policy debate. These key individuals can be described as water policy entrepreneurs who have succeeded in helping to push the climate adaptation issue on the regional water policy agenda. The identified policy entrepreneurs are almost exclusively senior civil servants from regional Dutch authorities such as Dutch waterboards and provinces. The policy entrepreneurs are found to use a variety of entrepreneurial strategies, framing ideas on climate adaptation to deal with different German and Dutch cross-border discourses, using venue shopping and creation to deal with differing institutional and organisational framework in Germany and the Netherlands, exploiting problem windows (such as floods) and political windows of opportunity (such as INTERREG) to advance and promote climate adaptation, and in managing cross-border networks, they employ specific ‘bridging strategies’ to deal with cultural differences and language barriers. Regarding implementation, we found that climate change and adaptation are mentioned in cross-border policy documents and preparatory studies, but are not yet translated in coordinated cross-border projects and programmes on the ground. The stage of policy implementation has not yet been reached, and the impact of policy entrepreneurs and their activities is found to have led to policy changes on paper, but not yet succeeding on the ground.

Moving beyond our original set of research questions, we want to reflect on four major challenges that cross-border entrepreneurs are encountering in the specific context of transboundary water management and which may contribute to the broader debate on innovative concepts of governance in climate adaptation.

### Cross-border challenges and specific entrepreneurial strategies in a cross-border setting

What are the particular challenges of cross-border entrepreneurship and specific entrepreneurial strategies needed to cope with differences between neighbouring countries? Four challenges are found to be of importance: international agenda setting, different institutional and organisational structures, cultural differences and different resource availability. These challenges will, to a certain extent, also be present in a purely domestic policy setting without international borders; however, they are exacerbated in cross-border cooperation.

The first challenge concerns the international agenda setting and bridging the difference in the policy debates in Germany and the Netherlands on climate change. Put shortly, the Dutch policy debate on the regional level has been strongly centred on climate adaptation, especially in the water sector (as evidenced by the Dutch Deltaprogramme and Space for the River programme) whereas in Germany, climate change is mostly discussed in terms of climate mitigation in the energy domain, moving towards renewable energies (as evidenced by the *Energiewendegesetz*). Different domestic policy styles and dynamics on climate change are playing out and are juxtaposed in the cross-border setting. Dutch policy entrepreneurs are faced with the task of promoting climate adaptation ideas and concepts with the German partners, thus actively engaging in the process of framing as well as policy innovation and diffusion to achieve goal congruency.

In this regard, we note that the detrimental effects of climate change still determine the general water policy debate; however, there are clear indications in recent policy documents concerning the study area (WRIJ 2015) that climate change is also posited as a chance, for example for intensified agricultural activities and larger agricultural output due to enhanced climatic conditions. This trend cannot only be referred to as a ‘blockade strategy’ to prevent further climate adaptation (and mitigation) measures but may fundamentally shift the debate on climate change at the regional scale and may deserve close following and further research.

The second challenge is posed by different institutional and organisational structures with different tasks, responsibilities and mandates. It is crucial to understand these differences in order to find the right counterparts and to build cross-border networks that last and can have an impact on the policy debate. In particular, it is necessary to understand which institutions and government levels are involved in policy formation and formulation on one hand, and policy implementation and execution on the other. In particular in Germany, responsibilities for policy formulation and implementation at the regional level are fragmented over four to five governmental levels alone (*Bundesebene*, *Landesebene*, *Bezirksregierungen*, *Kreise* and *Kommunen*). In the Netherlands, there are also four institutional layers in the water sector (Rijkswaterstaat, provinces, Municipalities and Waterboards). However, at the regional level, it is mainly the Dutch waterboards, which are responsible for both regional water policy formulation and implementation, and which do not have an institutional German equivalent in the study area. Cross-border policy entrepreneurs therefore need to create cross-border venues and networks that include German actors both at the very local level, for implementation, as well as the federal level, for policy formulation and planning. Carefully creating institutional and organisational arrangements, including process rules, is essential to successfully navigate the international, institutional landscape.

The third challenge concerns cultural differences and on how to connect actors across borders. Cross-border cooperation is not an abstract undertaking, but involves connecting individuals and building trust, confidence and personal relationships as a fundamental basis for cooperation while not sharing the same language and having different, though similar cultural background (Steers et al. 2013; Jacobs 2016). ‘Playing the cross-border policy game’ with a deep understanding of cultural peculiarities and differences differs from cooperating within one country and becomes thus a third decisive entrepreneurial tool, next to joint agenda setting and appropriate institutional arrangements.

Last but not least, we find a fourth challenge regarding the different availability of resources in both countries, of pivotal importance. Defining resources in broad terms as financial, human and legal resources (for example property rights), this challenge is the main obstacle moving from policy change on paper to policy change on the ground. While the (transactional) costs of governmental policy formulation on climate adaptation may be relatively low, implementation of climate adaptation measures in the water sector may involve considerable infrastructural investments and substantially infringe on property rights and land use of non-governmental stakeholders such as farmers. Especially, in the German part of the study area with intensive agricultural land use, this poses a major challenge for climate adaptation, in particular for relatively ‘radical’ approaches which require different land use. In addition, local and regional German water authorities have much less financial and human resources at their disposal than the Dutch waterboards, and subsequently much less clout and autonomy. The challenge of scarce resources is closely related to choosing appropriate institutional and organisational arrangements for implementation of policy measures as well as charting a course to find the necessary resources, for example through European INTERREG funding, as attempted and witnessed in the KARMA project.

Addressing these four challenges—cross-border agenda setting, choosing appropriate institutional cross-border arrangements, skilfully playing the cross-border policy game and addressing resource scarcity—is an essential exercise for cross-border policy entrepreneurs to take into account the domestic policy dynamics involved (Skjaerseth 2000) and to successfully steer the actors towards joint, cross-border action on climate adaptation.

The case study analysed here has not only demonstrated the importance of cross-border entrepreneurship in pushing climate adaptation on the regional cross-border policy agenda, while not obligated or catalysed by Dutch, German or European legislation such as the Water Framework Directive. Simultaneously, it showed the difficulties involved to move beyond climate adaptation in policy documents, policy change on paper, towards climate adaptation in practice and policy change on the ground.

Two suggestions are put forward for further research to look further into the emergence, activities and impact of cross-border policy entrepreneurs. First, we found a skewed distribution of entrepreneurship with most entrepreneurs at the regional and local level coming from the Netherlands. The limited geographical scope of this study does not allow a full comparative analysis to draw general inferences on German and Dutch entrepreneurship. For further research, it is therefore suggested to carry out a comprehensive Dutch-German comparative analysis of policy entrepreneurship on climate adaptation, explicitly including the emergence of entrepreneurial activities at the regional and national government level as well as the scientific community for a broader empirical basis.

Second, we focused on the study area of Deltarhine as the larger analytical unit, encompassing different venues of cross-border cooperation. Our understanding of policy entrepreneurship in Deltarhine can be further enhanced by carrying out venue-specific analyses and causally linking specific entrepreneurial strategies to policy outcomes for each venue. In addition, a comparative case study with other (European) border regions and regional cross-border river basins is suggested, which would also allow for a quantitative analysis of how different forms of policy entrepreneurship may cause policy change.

## Electronic supplementary material

ESM 1 (DOCX 66 kb)

## Footnotes

1 Netherlands: Waterboard Vechtstromen (prior to 2014 with its predecessors Waterboard Velt en Vecht and Waterboard Regge en Dinkel), Waterboard Rijn en IJssel, Province of Gelderland, Province of Overijssel, Ministry of Infrastructure and Environment. Germany: Landkreis Grafschaft Bentheim, Kreis Borken, Niedersächsische Landesbetrieb für Wasserwirtschaft, Küsten- und Naturschutz (NLWKN), Bezirksregierung Münster

2 Ecosystem-based Adaptation (EbA) is aimed at mitigating water shortage by using the natural buffer capacity, an ecosystem service of the landscape. Important components are river and floodplain restoration, surface water retention and increased infiltration rates on arable land.

3 The respective German and Dutch policy guidelines and programs at the national and federal state level can be accessed through the website of Climate-Adapt, the Climate Adaptation Platform of the European Commission.

4 See for example (Klijn et al. 2010) for detailed entrepreneurial strategies such as Process arrangements, Exploring content, Arranging and Connecting actors
