# Supplementary material for: Policy entrepreneurs in international river basins—getting climate adaptation on the cross-border water policy agenda
Source: Reg Environ Change. 2017 Jul 21;18(5):1287–98. doi: 10.1007/s10113-017-1178-5 (PMC6448351; doi:10.1007/s10113-017-1178-5)
Supplement: Supplementary file 1 — (DOCX 66 kb) [file 10113_2017_1178_MOESM1_ESM.docx]

*Regional Environmental Change*

Electronic Supplemental Information for

**Entrepreneurs in international river basins - how to get climate adaptation on the cross-border water policy agenda**

Enter authors here: T. Renner^1^, S. Meijerink^2^

^1^ Radboud University, Institute for Science, Innovation and Society, P.O. Box 9010, 6500 GL Nijmegen, The Netherlands, e-mail.: [tobias.renner@rhdhv.com](mailto:tobias.renner@rhdhv.com)

^2^ Radboud University, Institute for Management Research, Nijmegen, The Netherlands, e-mail: [s.meijerink@fm.ru.nl](mailto:s.meijerink@fm.ru.nl)

**Contents of this file**

Tables S1 to S3

**Introduction**

This SI file contains three supporting tables, containing a description of data sources and collected information:

- Table S1. Overview of data sources and information
- Table S2 Overview of literature pertaining to the study area and transboundary cooperation between Netherlands and Germany
- Table S3. Overview of respondents

Table S1. Overview of data sources and information

| **Archival records, minutes of meetings** |
| --- |
| - Minutes of Meetings of the Border Waters Commission (selected documents out of 8 meetings, 2005-2012) - Minutes of Meetings and reports of the subcommissions D (Berkel/Issel), E (Vechte-Dinkel) and F (Bourtanger Moor) of the Border Waters Commission (selected documents out of 10 meetings between 2005 -2012) - Minutes of Meetings and reports of the Deltarhine Steering Group (SGDR, 13 meetings, 2005-2014) and its Working Group (AGDR, 27 meetings, 2005-2014) - Minutes of Meetings, notes and reports of the Transboundary Platform for Regional Water Management - Steering Group (5 sessions, 2011-2015), its Strategic Working Group (16 meetings, 2011-2015) - Dutch-German treaties such as the Dutch-German Border Treaty of 1960 and its 12 sub-treaties (Grenzgewässervereinbarungen) pertaining to the study area (1973 to 1986) - International River Basin Management Plan Deltarhine 2009-2015 - Factsheets Cross-border Climate change and adaptation (2010-2014) - Operational Programme INTERREG V and Dutch-German position paper on importance climate adapation in the future INTERREG V programme |
| **Study reports and relevant policy documents** |
| Dutch policy documents based on Delta act   - Room of the River Vecht 2009-2014 - River Regge 2007-2009 - Freshwater programme East Netherlands (Zoetwatervoorziening Oost Nederland)   Dutch regional policy documents   - Watervision 2030 Waterboard Rijn en IJssel (Watervisie 2030) - Draft WFD management plan Rhine-East 2016-2021 (Ontwerp waterbeheerplan Rijn-Oost)   German policy documents Lower Saxony   - Draft WFD management plan 2016-2021   German policy documents Northrhine-Westphalia   - Draft WFD management plan 2016-2021   Cross-border policy documents   - Cross-border Vechtvision (Grensoverschrijdende Vechtvisie 2007-2009) - International RBMP Deltarhine 2009-2015 - Draft International RBMP Eems 2016-2021 - Archival records AGDR/SGDR – Factsheets Cross-border Climate change and adaptation (2010-2014) - Operational Programme INTERREG V and Dutch-German position paper on importance climate adapation in the future INTERREG V programme (2012)   Study reports and documents   - Strengthening cross-border Rhine dikes at the Dutch-German border - Cross-border restoration project Schoonebeekerdiep (2004-2014) - Draft Cross-border Interreg-project KARMA 2014 - Draft Cross-border project Climate adaptation in the Dutch-German Dinkel river (2014) - Cross-border project ‘Gewässerkonzept Schlinge‘ (2015)   Major climate change research projects   - Knowlegde for climate (KiK) – Netherlands - KLIMZUG, KLIFWA and Dynaklim – Germany |
| **Interviews** |
| Semi-structured interviews on cross-border cooperation were carried out in2013 and 2014 with main questions pertaining to a) the current and future policy challenges in cross-border cooperation relating to climate adaptation such as flood protection and water shortage future challenges, b) main drivers of cross-border cooperation in these areas, c) role and activities of key individuals, d) policy changes on paper and on the ground and e) contextual variables (c.q. institutional, organizational, cultural setting).  The interviews were open, semi-structured and held in German and Dutch to make it easier for the respondents to express nuances and detailed descriptions in their own language. In total, information of 20 interviews with respondents at the local and regional level were collected and used. Non-exhaustively, the interview partners from the following organizations were consulted:   - Netherlands, 10 respondents: Waterboards Velt en Vecht, Regge en Dinkel, Rijn en IJssel, provinces of Overijssel and Gelderland - Germany, 10 respondents: Kreis Borken, Landkreis Grafschaft Bentheim, Ministerium für Klimaschutz, Umwelt, Landwirtschaft, Natur- und Verbraucherschutz (MKULNV), Niedersächsische Landesbetrieb für Wasserwirtschaft, Küsten- und Naturschutz (NLWKN), Bezirksregierung Münster   See further Table S3 in the Supporting Information. |

Table S2. Overview of consulted literature pertaining to the study area and transboundary cooperation between Netherlands and Germany

| **Year** | **Title** | **Paper/ Article** | **Official document** | **Report** |
| --- | --- | --- | --- | --- |
| 1960 | Staatsvertrag (1960). Vertrag zwischen der Bundesrepublik Deutschland und dem Königreich der Niederlande zur Regelung von Grenzfragen und anderen zwischen beiden Ländern bestehenden Problemen (Ausgleichsvertrag) (1960). |  | **x** |  |
| 1978 | PGC-SGK (1978). Deutsch-niederländische Grenzgewässervereinbarungen 1963-1978. Bundesminister des Inneren. |  | **x** |  |
| 1981 | Mingst, K. A. (1981). The functionist and regime perspectives: The case of Rhine river cooperation. Journal of Common Market Studies 20: 161–173. | **x** |  |  |
| 1981 | Schutten, G.J. (1981). Varen waar geen water is. Geschiedenis van de scheepvaart ten oosten van de IJssel. Broekhuis, Hengelo. | **x** |  |  |
| 1988 | PGC-SGK (1988). 25 Jahre Ständige Deutsch-Niederländische Grenzgewässerkommission. Bundesministerium für Umwelt, Naturschutz und Reaktorsicherheit |  | **x** |  |
| 1992 | PGC-SGK - Unterausschuss VIIIIb Vechte (1992): Beheersplan Vecht. Plan zur Bewirtschaftung der Vecht. |  | **x** |  |
| 1993 | Dupont, C. (1993). The Rhine: A study of inland water negotiations. In Faure, G. & Rubin, J. (eds.): Culture and Negotiation: The resolution of water disputes, Thousand Oaks, 135-148. | **x** |  |  |
| 1993 | PGC-SGK - Subcommissies VII, VIIIA/B, IX (1993). Untersuchung der Gewässerqualität der deutsch-niederländischen Grenzgewässer 1977-1993. |  | **x** |  |
| 1993 | PGC-SGK (1993). Zusammenlegung von Unterausschüssen der Ständigen Grenzgewässerkommission. Anlage 1 zur Sitzungsprotokoll der 60. Sitzung der SGK. |  | **x** |  |
| 1993 | Soeters, J. (1993). Managing euregional networks. Organization Studies 1993 14: 639. | **x** |  |  |
| 1993 | Jansen, L.B. (1993). Bekend en onbemind - Het beeld van Duitsland en Duitsers onder jongeren van vijftien tot negentien jaar. Clingendael Institute, 's Gravenhage. |  |  | **x** |
| 1994 | Van der Goot, E.I. (1994). Gebied zonder grenzen? - grensoverschrijdende samenwerking in het Dinkeldal. MSc thesis. University of Twente, Almelo. |  |  | **x** |
| 1995 | Landkreis Grafschaft Bentheim, Provincie Overijssel (1995). Grenzüberschreitender Rahmenplan für Erholung und Tourismus im Vechtetal. |  |  | **x** |
| 1996 | Bernauer, T. (1996). Protecting the Rhine river against chloride pollution. In: R. O. Keohane and M. A. Levy (eds.), Institutions for Environmental Aid: Pitfalls and Promise, MIT Press, Cambridge, Mass., 201–233. | **x** |  |  |
| 1996 | Bernauer, T.; P. Moser, (1996). Reducing pollution of the river Rhine: The influence of international cooperation. Journal of Environment and Development, 5: 391–417. | **x** |  |  |
| 1996 | Staatliches Umweltamt Herten (1996). Gewässeruntersuchung im D-NL Stromgebiet der Berkel 1992-1995. |  |  | **x** |
| 1997 | PGC-SGK - Subcommissie Vecht en Dinkel (1997). 2e Interimrapport Beheersplan Vecht. |  | **x** |  |
| 1997 | Rijkswaterstaat (1997). De Vechtvisie: vrijheid in gebondenheid. |  | **x** |  |
| 1998 | Bezirksregierung Münster (1998). Auenentwicklungsprogramm NRW. Project Berkel. |  |  | **x** |
| 1998 | Gurtner-Zimmermann, A. (1998). The effectiveness of the Rhine action program. International Environmental Affairs 10:241–267. | **x** |  |  |
| 1998 | PGC-SGK (1998). Atlas van de Nederlands-Duitse grenswateren. |  | **x** |  |
| 1998 | Provinz Overijssel (1998) Conference Proceedings "Bewirtschaftungspläne im europäischen Raum: Beispiel Vechte". 27.5.1998. |  |  | **x** |
| 1998 | Warnecke, T.; Wilbrand, S. (1998). Probleme und Chancen grenzüberschreitender Zusammenarbeit am Beispiel des Hochwasserschutzes in der Dinkelaue. Studie Universität Dortmund, Fakultät Raumplanung. |  |  |  |
| 1998 | Waterschap Regge en Dinkel, StUA Herten (1998). Problematische Wasserinhaltsstoffe im Einzugsgebiet der Dinkel. |  |  | **x** |
| 2000 | PGC-SGK - Subcommissie Vecht en Dinkel (2000), werkgroep Waterkwaliteit. 1. Werkrapport Waterkwaliteit Vecht-Dinkel . |  | **x** |  |
| 2000 | Van Ast, J. (2000). Interactief Watermanagement in grensoverschrijdende riviersystemen. PhD Dissertation. Eburon, Delft. |  |  | **x** |
| 2000 | Verweij, M. (2000). Transboundary Environmental Problems and Cultural Theory: The Protection of the Rhine and the Great Lakes. Palgrave, Basingstoke UK and New York. | **x** |  |  |
| 2000 | Wielenga, F. (2000). Vom Feind zum Partner. Die Niederlande und Deutschland seit 1945. Agenda Verlag, Münster. | **x** |  |  |
| 2000 | Linthout, D. (2000) Onbekende buren. Atlas. | **x** |  |  |
| 2001 | Bezirksregierung Münster et.al. (2001). Grensoverschrijdende Dinkelplanning - Grenzüberschreitende Dinkelplanung. |  | **x** |  |
| 2003 | Jongman, R.H.G. (2003). Groene Band Hamaland.Wageningen, Alterra. Alterra rapport 743. |  |  | **x** |
| 2004 | Provincie Limburg (2004). Waterbeleid bij de buren. |  |  | **x** |
| 2004 | Schröder, R. et.al (2004). Regiodialoog Hamaland verslag atelier. Wageningen. Alterra rapport 938. |  |  | **x** |
| 2004 | Stichting de Derde Berkelcompagnie (2004). Berkelleitbild - Kontraste erleben an der Berkel. |  |  | **x** |
| 2004 | Van Slobbe, E. (2004). The Overijsselse Vecht in the Netherlands. SLIM project - case study monograph. |  |  | **x** |
| 2005 | Huitema, D. and Becker, G. (2005). Governance, institutions and participation: a comparative assessment of current conditions in selected countries in the Rhine, Amu Darya and Orange river basins. Report to the NEWATER project (D121), Institute for Environmental Studies - Vrije Universiteit Amsterdam. | **x** |  |  |
| 2005 | Unie van Waterschappen (2005). Geen brug te ver - Nota internationale zaken Unie van Waterschappen 2005-2010. |  |  |  |
| 2006 | Gemeente Oude IJsselstreek (2006). News bulletin "1. Deutsch-niederländische Wasserkonferenz Issel und Aastrang". 26.9.2013. Ulft. |  |  | **x** |
| 2006 | Keetman, W. (2006). Grensoverschrijdend Vechtwerk: op naar betere Duits-Nederlandse samenwerking met de KRW?. H2O 21/2006. | **x** |  |  |
| 2006 | Keetman, W. (2006). Grensoverschrijdende samenwerking in de regio. Unie van Waterschappen. | **x** |  |  |
| 2006 | Kreis Coesfeld (2006). Sitzungsvorlage "Grenzüberschreitendes Leitbild für die Berkel". |  |  | **x** |
| 2006 | Van der Molen, J. & H. Emmrich, 2006. Informeel contact als bouwsteen voor internationale samenwerking (interview). Het Waterschap, 2006(8), 12-13. | **x** |  |  |
| 2006 | Waterschap Velt en Vecht (2006). Brochure Schoonebeekerdiep. |  | **x** |  |
| 2006 | Rabobank (2006). Economic key indiactors Dutch-German border region. VNDU presentation. |  |  |  |
| 2007 | AcW/CAW, 2007. Bruggen bouwen; Nederlands waterbeheer in Europees en grensoverschrijdend perspectief. Gezamenlijk advies van de Adviescommissie Water (AcW) en de Commissie van advies inzake de waterstaatswetgeving (CAW) aan de Staatssecretaris van Verkeer en Waterstaat, 40 pp. |  | **x** |  |
| 2007 | Feld C.K.; Locker-Grutjen, O. (2007). River restoration in the IJssel catchment. In: Verwijmeren, J.; Wiering, M. (eds) Many rivers to cross—cross border co-operation in river management. Eburon, Delft, pp 49–70 | **x** |  |  |
| 2007 | Möllenkamp, S. (2007). The “WFD effect” on upstream downstream relations in international river basins - Insights from the Rhine and the Elbe basins, Hydrology and Earth System Sciences,4 , 1407-1428. | **x** |  |  |
| 2007 | Mostert, E. (2007). Internationale waterschappen? Nut, noodzaak en alternatieven. Report commissioned by AdviescommissieW ater. |  |  | **x** |
| 2007 | Provincie Overijssel (2007). Blauwe knooppunten deelstroomgebied Rijn-Oost. |  |  | **x** |
| 2007 | Renner, T.; Fiselier, J.; Heymans, J. (2007). ‘Grensoverschrijdend waterbeheer in Nederland’. DHV rapport in opdracht van Adviescommissie Water (ACW) en Commissie inzake Advies Waterstaatswetsgeving (CAW) (www.adviescommissiewater.nl) |  |  | **x** |
| 2007 | Rijkswaterstaat (2007). Uit de geschiedenis van de Overijsselsche Vecht. Unpublished manuscript. |  |  | **x** |
| 2007 | Van Leussen, W. et.al. (2007). Transboundary Governance and the Problem of Scale for the Implementation of the European Water Framework Directive at the Dutch-German Border. | **x** |  |  |
| 2007 | Verwijmeren, J.; Wiering, M. (2007). Many rivers to cross. Eburon, The Netherlands. | **x** |  |  |
| 2007 | Waterschap Velt en Vecht (2007). Cross-border application of the Blue Knots concept at Waterboard Velt en Vecht. |  |  | **x** |
| 2008 | Mostert, E. (2008). International cooperation on Rhine water quality 1945-2008: an example to follow? Physics and Chemistry of the Earth. | **x** |  |  |
| 2008 | Renner, T. et.al. (2008) Inventarisatie Grensoverschrijdende Vechtvisie. DHV rapport in opdracht van Waterschap Velt en Vecht. (www.devecht.eu) |  |  | **x** |
| 2008 | Stichting Verbetering Oude Ijsselzone (2008). Visie Oude Ijsselzone - Toekomst voor een verborgen rivier. | **x** |  |  |
| 2009 | Gilissen, H.K. (2009). Internationale en regionaal grensoverschrijdende samenwerking in het waterbeheer. Sdu Uitgevers, Den Haag. | **x** |  |  |
| 2009 | Havekes, H. (2009). Functioneel Decentraal Waterbestuur: Borging, Bescherming en Beweging. PhD Thesis, Van Hall Institute. | **x** |  |  |
| 2009 | Kreis Borken (2009). Werkconferentie "Goud in de grond" 9.11.2009. Lievelde. |  |  | **x** |
| 2009 | Renner, T. et.al. (2009) Grensoverschrijdende Vechtvisie - Grenzüberschreitende Vechtetalstrategie. DHV rapport in opdracht van Waterschap Velt en Vecht. (www.devecht.eu) |  |  | **x** |
| 2009 | Waterschap Rijn en IJssel (2008). Statistische analyse waterkwaliteit in beheersgebied waterschap Rijn en IJssel 1982-2007. |  |  | **x** |
| 2009 | Waterschap Rijn en IJssel (2009). Evaluatie van 23 jaar macrofauna-monitoring bij Waterschap Rijn en IJssel 1987-2009. Internal report. |  |  | **x** |
| 2009 | Waterschap Velt en Vecht et.al. (2009). Grensoverschrijdende Vechtvisie - Grenzüberschreitende Vechtetalstrategie. |  | **x** |  |
| 2009 | Wiering, M. et.al. (2009). Experiences in regional cross border co-operation in river management. Comparing three cases at the Dutch-German border. Water Resources Management. | **x** |  |  |
| 2010 | Provincie Overijssel (2010). Cultuurhistorische Atlas van de Vecht. W Books. | **x** |  |  |
| 2010 | Raad voor Openbaar Bestuur (2010). Grensoverschrijdende samenwerking: terugblik en aanbevelingen. ROB rapport. |  |  | **x** |
| 2010 | Unie van Waterschappen (2010). Conference proceedings "Internationale KRW conferentie - Schoon water zonder grenzen". 26.1.2010, Middelburg. |  |  | **x** |
| 2010 | Van der Molen, J.; Lulofs, K. (2010). Guidance schemes for the boundary spanner, in: Hans Bressers and Kris Lulofs (eds.) Governance and complexity in Water Management. Creating Cooperation through Boundary Spanning Strategies. | **x** |  |  |
| 2010 | Waterboard Rijn en IJssel (2010). Conference proceedings "2. Deutsch-niederländische Wasserkonferenz Issel und Aastrang" 15.4.2010. Isselburg. |  |  | **x** |
| 2010 | Waterschap Velt en Vecht (2010). Brochure Flow in het Vechtdal. |  |  |  |
| 2010 | Duitslandinstituut (2010). Rapport Belevingsonderzoek Duits 1993-2010. |  |  | **x** |
| 2010 | Wester et.al. (2010). Belangrijke buur, blinde vlek. Onderzoek naar ontwikkelingen in de relatie tussen Nederland en Duitsland op het gebied van onderwijs, cultuur en wetenschap. Berenschot, commissioned by the Dutch Ministry of Education. |  |  | **x** |
| 2011 | Mininsterie voor Infrastructuur en Milieu (2011). Conference proceedings "Haarmühlesymposium - Grensoverschrijdend samenwerken water-natuur-ruimtelijke ordening". 22.6.2011, Haarmühle. |  |  | **x** |
| 2011 | Saxion Hogescholen Enschede (2011). Conference proceedings "20 Jahre Vertrag von Anholt". 25.5.2011, Wasserburg Anholt. |  |  | **x** |
| 2011 | Van der Molen, J. (2011). Crossing Borders: Een kader voor het tot ontwikkeling brengen van grensoverschrijdende samenwerking in watermanagement. Waterschap Velt en Vecht, Coevorden. | **x** |  |  |
| 2011 | Van Herten, M. (2011). Bridging the Dutch-German border - network analysis Rhine-East. Honours Thesis, Utrecht University | **x** |  |  |
| 2011 | Waterschap Velt en Vecht (2011). Conference proceedings "2e Duits-Nederlands Vechtsymposium" 8.9.2011. Schöppingen. |  |  | **x** |
| 2012 | Bezirksregierung Münster et.al (2012). Abschlussbericht Grenzüberschreitender Hochwasserschutz. March 2012 |  | **x** |  |
| 2012 | Van der Meulen, S. et al (2012): Towards practical implementation of the ecosystem services (ES) concept in transboundary water management. Project report Deltares. Delft. |  |  |  |
| 2012 | Van der Molen, J.; Ietswaart, H. (2012). Crossing Borders: Practical handbook to the Crossing Borders theory. | **x** |  |  |
| 2012 | Wiering, M.; Verwijmeren, J. (2012): Limits and Borders: Stages of Transboundary Water Management, Journal of Borderlands Studies, 27:3, 257-272 | **x** |  |  |
| 2012 | Onderzoeksbureau Atlas voor gemeenten (2012). Atlas van kansen voor de grensregio Achterhoek-Borken. Report, commissioned by Ministerie van BZK. | **x** |  |  |
| 2013 | PGC-SGK (2013). Conference proceedings "50 Jahre Ständige Deutsch-Niederländische Grenzgewässerkommission". Bundesministerium für Umwelt, Naturschutz und Reaktorsicherheit |  | **x** |  |
| 2013 | Stichting de Derde Berkelcompagnie (2013). Conference proceedings 5e Berkelconferentie 14.2.2013. Vreden. |  |  | **x** |
| 2013 | Waterschap Rijn en IJssel (2013). Position paper Interreg Va "Samenwerking in het waterbeheer langs de Duits-Nederlandse grens". |  |  | **x** |
| 2013 | Lak, M. (2013). Status quo und Zukunft der grenzüberschreitenden Beziehungen Niederlande - Deutschland. Vortrag vom 17. Januar 2013 im Rahmen einer Vorlesungsreihe am Zentrum für Niederlande-Studien der Westfälischen Wilhelms-Universität Münster. | **x** |  |  |
| 2013 | Pekelder, J. (2013). Neue Nachbarschaft - Deutschland und die Niederlande, Bildformung und Beziehungen. Agenda Verlag, Münster. | **x** |  |  |
| 2014 | Bezirksregierung Münster, Waterboard Rijn en IJssel (2014). Grenzüberschreitendes Gewässerkonzept Schlinge/Bovenslinge. |  |  | **x** |
| 2014 | Bundesministerium für Umwelt, Naturschutz Bonn Naturschutz, Bau und Reaktorsicherheit (2014). Ergebnisvermerk Gespräch zur zukünftigen Ausrichtung der Ständigen Deutsch-Niederländischen Grenzgewässerkommission am 27. Mai 2014 im MKULNV, Düsseldorf. |  | **x** |  |
| 2014 | Kreis Borken (2012). Pilotprojekt Schlinge - Minimierung diffuser Nährstoffeinträge in Oberflächengewässer im Projektgebiet Oeding-Winterswijk. |  |  | **x** |
| 2014 | Kreis Borken (2014). Planungsmassnahmen Hochwasserschutzkonzept Issel. |  |  | **x** |
| 2014 | Stichting Derde Berkelcompagnie (2014). News bulletin 'Nieuwsquelle" 2005-2014. |  |  | **x** |
| 2014 | Waterschap Vechtstromen (2014). News bulletin 'Stopping project Schoonebeekerdiep'. |  | **x** |  |
| 2014 | Waterschap Vechtstromen (2014). Trendanalyse waterkwaliteit 1990-2010. Internal report, unpublished. |  |  |  |
| 2014 | Van der Giessen, M. (2014). Coping with complexity. Cross-border cooperation between the Netherlands and Germany. PhD thesis Radboud University. | **x** |  |  |
| 2014 | Van Eerd, M.; Wiering, M.; Dieperink, C. (2014). Exploring the Prospects for Cross-Border Climate Change Adaptation between North Rhine-Westphalia and the Netherlands. Utrecht Law Review, Volume 10, Issue 2 (May) 2014. | **x** |  |  |
| 1963-2014 | PGC-SGK (1963-2014). Minutes of Meetings 1st meeting on 2/3.12.1963 until 80th meeting |  | **x** |  |
| 1993-2005 | PGC-SGK - Subcommissie Bourtanger Veen (1993-2005). Minutes of Meetings. |  | **x** |  |
| 1993-2005 | PGC-SGK - Subcommissie Vecht en Dinkel (1981-1998). Jaarverslagen Waterkwaliteit Dinkel, Overijsselsche Vecht en zijbeken. |  | **x** |  |
| 1993-2005 | PGC-SGK - Subcommissie Vecht en Dinkel (1993-2003). Minutes of meeting. 1st meeting on 2.6.1993 until 2003 |  | **x** |  |
| 1993-2014 | PGC-SGK - Subcommissie VII, VIIIA en VIIIB (1988-1992). Minutes of meeting. 24/26st meeting on 10.1.1988 until 27.5.1992 |  | **x** |  |
| 1993-2014 | PGC-SGK - Subcommissie Berkel/Oude IJssel (1993-2008). Minutes of meeting. 1st meeting on 7.6.1993 until 29.5.2008 |  | **x** |  |
| 1995-2002 | PGC-SGK - Subcommissie Berkel/Oude IJssel (1995-2002). Jaarverslagen waterkwaliteit grenswateren Gelderland/NRW. |  | **x** |  |
| 2005-2014 | Internationale Arbeitsgruppe Deltarhein (2005-2013). Minutes of Meetings. 1st meeting on 9-5-2005 until 27th meeting on 24-9-2014. |  |  |  |
| 2005-2014 | Internationale Steuerungsgruppe Deltarhein (2005-2013). Minutes of Meetings. 1st meeting on 18-1-2005 until 13th meeting on 21-5-2014. |  | **x** |  |
| 2007a | Van der Molen, J. & H. Emmrich, 2007a.. Grensoverschrijdend samenwerken bij het beheer van de Vecht: onderweg naar morgen. Het Waterschap, 2007(6), 22-25. | **x** |  |  |
| 2012-2014 | GPRW (Grenzüberschreitende Plattform für Regionale Wasserwirtschaft) (2012-2014). Strategieteamtreffen - Minutes of Meetings. 1st meeting on 17-4-2012 until 20-11-2014. |  | **x** |  |
| 2012-2014 | GPRW (Grenzüberschreitende Plattform für Regionale Wasserwirtschaft) (2012-2014). Vorstandstreffen - Minutes of Meetings. 1st meeting on 5-7-2012 until 23-6-2014. |  | **x** |  |

Table S3. Overview of respondents

| **Year** | **Organisation** | **Germany** | **Netherlands** | **Record** |
| --- | --- | --- | --- | --- |
| 2013 | Waterschap Rijn en IJssel |  | X | 8-1-2013 |
| 2013 | Waterschap Rijn en IJssel |  | X | 8-1-2013 |
| 2014 | Waterschap Regge en Dinkel |  | X | 17-2-2014 |
| 2014 | Provincie Overijssel |  | X | 26-2-2014 |
| 2014 | Waterschap Rijn en IJssel |  | X | 12-2-2014 |
| 2014 | Waterschap Vechtstromen |  | X | 26-2-2014 |
| 2014 | Waterschap Vechtstromen |  | X | 7-5-2014 |
| 2014 | Ministerie van Infrastructuur en Milieu |  | X | 21-5-2014 |
| 2014 | Provincie Overijssel |  | X | 3-6-2014 |
| 2014 | Waterschap Vechtstromen |  | X | 12-6-2014 |
| 2014 | Provincie Gelderland |  | X | 3-7-2014 |
| 2013 | Landkreis Grafschaft Bentheim, | X |  | 10-1-2013 |
| 2013 | Kreis Borken | X |  | 5-7-2013 |
| 2013 | Landkreis Grafschaft Bentheim, | X |  | 10-1-2013 |
| 2014 | NLWKN | X |  | 24-2-2014 |
| 2014 | Ministerium für Klimaschutz, Umwelt, Landwirtschaft, Natur- und Verbraucherschutz (MKULNV) | X |  | 21-5-2014 |
| 2014 | Niedersächsische Landesbetrieb für Wasserwirtschaft, Küsten- und Naturschutz (NLWKN) | X |  | 12-2-2014 |
| 2014 | Bezirksregierung Münster | X |  | 5-6-2014 |
| 2014 | Landkreis Grafschaft Bentheim, | X |  | 17-6-2014 |
| 2014 | Landkreis Grafschaft Bentheim, | X |  | 17-6-2014 |
| 2014 | Bezirksregierung Münster | X |  | 12-11-2014 |
| 2014 | Kreis Borken | X |  | 22-4-2014 |
